# Making patient centered care a reality: a survey of patient educational programs in Italian Cancer Research and Care Institutes

**DOI:** 10.1186/s12913-015-0962-5

**Published:** 2015-07-30

**Authors:** C. Cipolat Mis, I. Truccolo, V. Ravaioli, S. Cocchi, L. Gangeri, P. Mosconi, C. Drace, L. Pomicino, A. Paradiso, P. De Paoli

**Affiliations:** Scientific and Patients Library, CRO National Cancer Institute - IRCCS, Aviano (PN), Italy; Public Relations, Communication and Press Office, Istituto Scientifico Romagnolo per lo Studio e la Cura dei Tumori (IRST) Srl IRCCS, Meldola, Italy; Medical Library, IRCCS-Arcispedale Santa Maria Nuova, Reggio Emilia, Italy; Clinical Psychology Unit, Fondazione IRCCS Istituto Nazionale dei Tumori, Milano, Italy; Laboratory for medical research and consumer involvement, IRCCS-Istituto di Ricerche Farmacologiche Mario Negri, Milano, Italy; Familiar Cancer Clinic, Veneto Institute of Oncology IOV – IRCCS, Padova, Italy; Department of Pediatrics, Institute of Maternal and Child Health, Burlo Garofolo, Trieste, Italy; Experimental Medical Oncology Unit, National Cancer Research Centre, Istituto Tumori’Giovanni Paolo II’, Bari, Italy; Scientific Director, CRO National Cancer Institute - IRCCS, Aviano (PN), Italy

**Keywords:** Patient education, Neoplasms, Comprehensive cancer centers, Patient participation, Data collection, Surveys, Educational policies, Educational activities, Healthcare workers

## Abstract

**Background:**

Educational intervention represents an essential element of care for cancer patients; while several single institutions develop their own patient education (PE) programs on cancer, little information is available on the effective existence of PE programs at the level of research and care institutes. In Italy such institutes - Istituti di Ricovero e Cura a Carattere Scientifico - are appointed by the Ministry of Health, and 11 (Cancer Research & Care Istitute-CRCI) of the 48 are specific for cancer on the basis of specific requirements regarding cancer care, research and education. Therefore, they represent an ideal and homogeneous model through which to investigate PE policies and activities throughout the country. The objective of this study was to assess PE activities in Italian CRCI.

**Methods:**

We carried out a survey on PE strategies and services through a questionnaire. Four key points were investigated: a) PE as a cancer care priority, b) activities that are routinely part of PE, c) real involvement of the patients, and d) involvement of healthcare workers in PE activities.

**Results:**

Most CRCI (85 %) completed the survey. All reported having ongoing PE activities, and 4 of the 11 considered PE an institutional activity. More than 90 % of CRCI organize classes and prepare PE handouts, while other PE activities (e.g., Cancer Information Services, mutual support groups) are less frequently part of institutional PE programs. Patients are frequently involved in the organization and preparation of educational activities on the basis of their own needs. Various PE activities are carried out for caregivers in 8 (73 %) out of 11 institutes. Finally, health care workers have an active role in the organization of PE programs, although nurses take part in these activities in only half of CRCI and pharmacists are seldom included.

**Conclusions:**

The information arising from our research constitutes a necessary framework to identify areas of development and to design new strategies and standards to disseminate the culture of PE. This may ultimately help and stimulate the establishment of institutional integrated PE programs, including policies and interventions that can benefit a significant proportion of cancer patients.

**Electronic supplementary material:**

The online version of this article (doi:10.1186/s12913-015-0962-5) contains supplementary material, which is available to authorized users.

## Background

Educational interventions for people with cancer constitute essential activities for cancer research and care institute (CRCI) [1–3]. These interventions are commonly defined as “Patient education” (PE) but in the field of oncology, there is no consensus about this concept, being a wide range of activities and experiences. Moreover, this concept has undergone many changes in the last 50 years, whether in theory, model, policy or practice [4, 5]. The evolution followed the transformations of the different socio-cultural contexts and changes in the care of patients: some interesting overviews of the transformation in the different countries are reported in the literature [6]. It is possible recognize at least three phases in the evolution of the PE concept over the last decades: a progressive major engagement of the patient in the healthcare organization and in the decision making process [7–11]; and, finally, the complementary role of the caregiver in heath care, in particular for chronic disease, as cancer is considered [12].

While these aspects are well covered in the international literature, less is known about the role of the health care organization in planning, implementing, monitoring and evaluating PE programs [12–16].

Since the implementation of a PE program is a big challenge for CRCI, in many cases, PE programs are not a part of institutional goals and strategies, but rather are developed in an uncoordinated fashion by clinical departments, single units or patient advocacy groups [17]. According to the Cancer Patient Education Network (CPEN), the biggest challenge for institutional PE programs is the commitment of the organizations’ leaders, including the establishment of PE as an organizational priority, the standardization of processes and an ensured access to adequate resources [18]. It has also been hypothesized that transforming the approach to educating cancer patients must overcome important barriers, such as the lack of awareness and adequate skills [19]. Furthermore, the participation of patients is essential for the successful development of effective research programs that result in the promotion of their health [20, 21]. Information regarding the above-mentioned issues, such as the active role of cancer patients in the realization of PE programs, is presently lacking. The analysis of PE programs and opportunities in oncology have mostly been limited to single institutions while national surveys have seldom been reported [6]. This type of analysis may be better suited for screening institutional attitudes in the delivery of care.

In the framework of the Italian National Health System, CRCI are officially appointed by the Ministry of Health according to specific requirements, including the ability to develop state of the art cancer care, research and education [22, 23]. Italian CRCI have been established nationwide and cure several hundred-thousand cancer patients every year. For this reason, they represent an ideal and homogeneous setting to investigate PE policies and attitudes in the oncology field throughout the entire country.

This survey was part of the 2010 research project “Extending Comprehensive Cancer Centers’ Expertise in patient education: the power of partnership with patient representatives” supported by the Italian Ministry of Health. The aim of this project is to increase the educational competence of a large number cancer patients, leading to an improved health status. This will also be achieved with the establishment of coordinated PE programs. The objective of the survey was to assess the situation regarding PE activity in Italian CRCI; in particular, we were interested in four key points: a) the presence or absence of PE as a cancer care priority, b) what activities are routinely part of PE, c) the active involvement of patient representatives in PE programs, and, finally, d) the role of health care workers (HCWs) in PE programs.

## Methods

Our survey aims to take a picture of the PE activities and processes in the CRCI through a PE questionnaire addressed to the Scientific Board Director.

### The survey

The PE questionnaire was mailed to the Scientific Board Director of all 13 CRCI (including one pharmacological CRCI with particular expertise in patient empowerment [24] and one pediatric CRCI with oncology unit). Hereinafter these institutions are referred to as Italian Cancer Patient Education Group – ICPEG, since all declared to have PE activities. The name and the contact information of the Scientific Board Director were obtained from the Italian Ministry of Health website. The Scientific Board Director of each CRCI was contacted in advance to be informed about the project and invited to participate in the survey. The PE questionnaire was sent via e-mail, during the first week of February 2013, with the request that it should be returned filled out, through the same means, by 20 February 2013. A letter signed by principal investigator of the research project (CRO Aviano) explained the goal of the project, the aim of the survey and the significance of the concepts included in the PE questionnaire. The letter also indicated that the participation did not require more than 20 min. Each Scientific Board Director had the possibility to delegate the compilation to another operator. Only one reminder was sent after 30 days.

### The PE questionnaire

An appropriate literature search was conducted to find a validated questionnaire in the PE field to be administered to the Scientific Board Directors. As no tools matched the purpose of the survey, the Centro di Riferimento Oncologico (CRO) of Aviano, as leader of the project and in collaboration with project partners, developed, an hoc questionnaire on the basis of:

- a previous OECI-Organization of European Cancer Institutes [25] questionnaire addressed to survey the ongoing activities of PE in the European cancer organizations;
- the results of a survey customer satisfaction/information needs assessment regarding the educational activities organized at CRO [26]
- the collaboration with the Princess Margaret Cancer Centre and its PE Programme [27, 28] and a professional relationship with the Cancer Patient Education Network [29].

The PE questionnaire was designed to detect the type of activity, the method of organization, the characteristics of persons involved in the different phases of each activity - plan-do-get feedback and revise -, and the extent to which the patients and/or their representatives are involved in the educational activities. Drafts of the questionnaire and the list of PE activities to be considered were shared during several online brainstorming with the other partners of the project. After feedback and review, a final consensus on the areas to be considered was reached. The final draft was finally discussed with the advisory board of the project and with some experts of PE in Italy [30].

At the end the PE questionnaire was organized into four sections: a) PE as a cancer priority in CRCI, b) organizational structure of PE activities, c) active involvement of patients/caregivers, and d) involvement of HCWs. In detail, the survey was structured in two parts with 19 main items: an initial description of the Institute (9 questions) and another part used to detect the various PE activities. Almost all questions were either yes/no or multiple choice. The only open answer questions referred to the description of the institute and the elaboration of the answer “other” in the multiple choice questions.

PE activities were defined as follows: 1) Classes: meetings with patients and caregivers on cancer topics held by healthcare personnel [31]; 2) PE handouts, i.e., “Works consisting of a handout or self-contained informative material used to explain a procedure or condition or the contents of a specific article in a biomedical journal and written in non-technical language for the patient or consumer” (MeSH/Medline definition) [32, 33]; 3) Cancer Information Services (CIS) that offer information via free and confidential one-on-one interaction with trained information specialists about cancer and support services to cancer patients, their family and friends, the public and healthcare professionals (http://icisg.org/) (e.g., Patient Library) [26, 34–37]; 4) educational programs for HCWs on specific issues such as communication, relationship with the patient, narrative medicine, etc. [38]; 5) wellness classes (e.g., gymnastics, cooking activities, meditation lessons, make-up, etc. [39]); 6) support services for caregivers (i.e., family members, relatives, caregivers) [12]; 7) the presence and organization of groups of mutual support [40]; and 8) presence and activities of volunteer associations in the Institute and active involvement of patient advocacies [41]. Copy of the questionnaire is reported in Additional file 1.

### Statistical analysis

We conducted a descriptive analysis of the survey, presenting data as percentages of responding ICPEG. We coded and processed the data from the returned surveys with the SAS program. Due to the reduced number of participants in the survey, no significant statistical tests have been applied. We only used frequency data to describe the ongoing PE activities in participating centers.

The Ethics Committee of the CRO approved the project and the survey.

## Results

We received 11 questionnaires out of 13 (85 %), as 2 centers did not send their completed questionnaire back. Seven of the respondents (63 %) were from Northern Italy, 2 (18 %) from Central Italy and 2 (18 %) from Southern Italy. When respondents did not answer all the items, the number of responders is reported in each table. We herein report the results regarding the four key points included in the questionnaire. The result is a “picture” of the patient education activities in the CRCI as described by their Scientific Board Director at the kickoff of the multicenter collaborative project.

### PE as a cancer-care priority in CRCI

This point investigated whether PE is part of the mission of ICPEG based on the fact that the Scientific Board Director or their delegates coordinated PE institutional policies. All participants developed PE activities, but only 4 of 11 (36 %) recognized PE as part of an institutional strategy shared by all the members of the organization, while in the other 7 ICPEG members (64 %), single units of the institution (i.e., medical oncology departments or medical libraries, associations of patients, etc.) performed PE activities. One institute had a written document that described, on an annual basis, the ongoing PE activities.

### Organizational structure of PE activities

This key point investigated the presence of specific PE activities that are routinely part of PE programs (Table 1). More than 90 % of ICPEG institutes organize classes, produce PE handouts and have partnerships with volunteer associations/patient advocacies, while the other PE activities are less frequently part of institutional PE programs.

**Table 1:** Patient education activities provided in Italian Cancer Patient Education Group - ICPEG (^a^)

	Type of Patient Education activities							
	Classes	PE handout	CIS (Cancer Information Service)	Continuing learning	Wellness classes	Support for caregivers	Groups of mutual support	Patient advocacy
Yes	10 (91 %)	10 (91 %)	7 (64 %)	9 (82 %)	9 (82 %)	8 (73 %)	8 (73 %)	10 (91 %)
No	1 (9 %)	1 (9 %)	4 (36 %)	2 (18 %)	2 (18 %)	3 (27 %)	3 (27 %)	1 (9 %)

^a^9 cancer research & care institutes, 1 pharmacological research & care institute with particular expertise in patient empowerment and 1 pediatric research & care institute with oncology unit

### Active involvement of patients/caregivers

Our survey investigated aspects regarding the active role of patients in PE programs; we will focus here on the established term “patient involvement” to denote the involvement of patients in activities related to planning and implementing PE activities [42]. First, we asked whether PE activities are based on patients’ needs; secondly, we investigated the effective participation of patients in the organization of the various activities.

The data displayed in Table 2 suggest that classes and patient education handouts are usually provided, taking into account the real needs of the patients, while the organization of CIS and wellness activities are less frequently based on patients’ needs. Our data shows that 75 % of ICPEG members support activities for caregivers that are based on their needs.

**Table 2:** Patient education activities based on patients' needs (^a^)

	Type of Patient Education activities						
	Classes	PE handout	CIS (Cancer Information Service)	Continuing learning	Wellness classes	Caregiver support	Groups of mutual support
Yes	8 (80 %)	7 (70 %)	6 (86 %)	4 (45 %)	6 (66 %)	6 (75 %)	4 (50 %)
No	2 (20 %)	3 (30 %)	1 (14 %)	5 (55 %)	3 (34 %)	2 (25 %)	4 (50 %)

^a^the total of each column/type of PE activity, is different according to the presence or absence of the activity in the institute, corresponding to the ICPEG institute which answered yes in the Table 1

Table 3 shows that patients are frequently involved in the organization of meetings on cancer topics and in the preparation of PE handouts while they are less frequently involved in the organization of wellness classes and mutual support groups.

**Table 3:** Involvement of patients in the preparation/organisation of the patient education activities (^a^)

	Type of Patient Education activities						
	Classes (present in 10 institutes)	PE handout (present in 10 institutes)	CIS (Cancer Information Service) (present in 7 institutes)	Continuing learning (present in 9 institutes)	Wellness classes (present in 9 institutes)	Caregiver support (present in 8 institutes)	Groups of mutual support (present in 8 institutes)
Yes	6 (60 %)	7 (70 %)	n.a.	3 (33 %)	3 (33 %)	3 (37 %)	3 (37 %)
No	4 (40 %)	3 (30 %)	n.a.	6 (67 %)	6 (67 %)	5 (63 %)	5 (63 %)

^a^The total of each column/type of PE activity, is different according to the presence or absence of the activity in the institute, corresponding to the ICPEG institute which answered yes in the Table 1

Regarding caregivers, 8 (73 %) out of 11 respondents reported some activities such as training sessions on disease and treatment in 6 institutes (75 %), and training sessions on homecare, training sessions on psychological support and other session, upon patients ‘families’ request, are activated in 2 institutes (25 %).

Regarding the activities reserved for caregivers, the majority of the responders - 8 out of 11 (73 %) – stated that there are some institutional activities addressed to caregivers; these are based on a preliminary survey, about 5 (62 %) out of 8 institutes respondent. These activities regard educational courses and/or training sessions addressed to the caregivers, both inside and outside the Institute (Table 4). Many HCWs are involved in these educational activities, both in the planning phase and implementation, with psychologists being the most involved.

**Table 4:** Activities for caregivers-family education (^a^)

Training sessions on disease and treatment	6 (65 %)
Training sessions on homecare	2 (25 %)
Training session on psychological support	2 (25 %)
other, on patients’ family request	2 (25 %)

(^a^) Respondents could select one or more of the items; therefore, the sum of each column may be > 100 %

Regarding the diffusion of these activities addressed to the caregivers, it emerges from the survey that only volunteers and HCWs are engaged in promoting the participation. It seems that no other tools are used for promotion despite the many complementary tools used for diffusing the other kind of PE activities, such as newsletters, press releases, website, social networks etc.…

### Involvement of HCWs

Table 5 shows what types of HCWs are involved in the organization of PE activities. The survey data shows that the majority of HCWs have an active role in the organization of PE programs, although nurses take part in these activities in only half of ICPEG members, and pharmacists are seldom included. Psychologists seem to be uninvolved in the organization of classes, while they are very active in all other activities. Medical doctors take part in the organization of educational programs for patient education in almost 80 % of institutes, while nurses and pharmacists less frequently.

**Table 5:** Involvement of health care workers in the organization phase of patient education program

	Type of Patient Education activities^a^				
Type of personnel	Classes (present in 10 institutes)	Continuing learning (present in 9 institutes)	Wellness classes (present in 9 institutes)	Caregiver support (present in 8 institutes)	Groups of mutual support (present in 8 institutes)
Administratives	8 (80 %)	0	1 (11 %)	1 (12 %)	0
Doctors	6 (60 %)	7 (78 %)	4 (44 %)	5 (62 %)	2 (25 %)
Nurses	5 (50 %)	4 (44 %)	3 (33 %)	5 (62 %)	2 (25 %)
Librarians	3 (30 %)	2 (22 %)	0	0	0
Pharmacists	2 (20 %)	0	0	0	0
Psychologists	0	7 (78 %)	8 (89 %)	7 (87 %)	4 (50 %)
Trainig office	0	9 (100 %)	0	0	0
Communication office	0	2 (22 %)	0	0	0
Others (external experts)	2 (20 %)	0	6 (67 %)	1 (12 %)	0

^a^Respondents could select part or all the items; therefore, the sum of each column may be > 100 %

## Discussion

PE activities oriented towards empowerment are becoming increasingly important for cancer care [24, 42, 43]. Important institutions such as the Institute of Medicine of the National Academies, USA, has recently strongly recommended providing cancer patients with understandable information on several aspects of cancer care and research [44]. As the results of this survey show, PE activities involve both patients and/or caregivers and/or HCWs and the relationship among them and the HCWs’ perception of the illness [45]. This is aligned with the international literature and the CPEN standards [CPEN standards]. But, while PE activities have been described in some institutions treating cancer patients [46, 47], very few studies have investigated the PE situation in a wider and more homogeneous context, such as comprehensive cancer centers nationwide. Recently, Italian CRCIs have started the process of accreditation for excellence based on standards developed by the Organization of European Cancer Institutes (OECI) [25] (www.oeci.eu), dealing with the accountability and involvement of the entire organization by adopting common, basic principles that can dictate further actions [23]. A section of the OECI standards collects general information on educational material; our idea was that obtaining more information on PE activities may be useful for designing interventional measures that may have an impact on the quality of cancer care. For this purpose, we carried out this survey to “photograph” the state of the art of PE activities in the CRCI as described by their organizations’ board at the kickoff of the project. Since the delivery of high-quality cancer care represents a goal of CRCI, it is particularly important to understand whether and how PE activities are realized throughout these institutions at the national level. The data from our survey demonstrated that Italian cancer centers offer access to educational activities; in the majority of cases, PE activities are not shared by all members of the organization, but are rather realized by single units of the institutions or by patient representatives on a volunteer basis. These data suggest that the organizational structure and culture of PE need to be further improved by the establishment of patient education as a cancer-care priority shared by the leadership and all members of the organization.

It has been suggested that the tools used to deliver education may have great influence on the results of PE programs [48]. Our survey demonstrates that participants use a combination of different approaches and tools to improve PE such as classes, PE handouts, cancer information services and mutual support groups. In our opinion, this extensive approach may facilitate meaningful contacts among participants resulting in a more effective patient-centered approach. To obtain additional information, our project includes a specific work package investigating the use of traditional and new tools in the delivery of educational materials in ICPEG members.

Furthermore, we have demonstrated that in 80 % of the centers responding to our questionnaire, PE activities were performed on the basis of patients’ needs and that in the majority of ICPEG, patients take an active role in the preparation and organization of PE. Seventy-three percent of responding centers organized, among others, specific activities for caregivers of oncological patients. Considering that caregivers showed improper recognition of the real needs for information of their own patients, these activities may be particularly important [49].

Finally, our data suggest that almost all the professionals involved in cancer care have an active role in the organization of PE activities, with the notable exception of pharmacists, who only in a minority of cases participate in classes. With the increasing understanding and recognition of drug interactions and side-effects, pharmacists can provide timely interventions and information to health providers, as well as counseling to patients [50]; for this reason, we strongly suggest that PE teams in Italian CRCI must necessarily include a pharmacist with expertise in oncology.

Since 2009, CRO has been collaborating in the field of patient education with the *Princess Margaret* Cancer Centre based in Toronto, one of the most important scientific research centre and teaching *hospital* in the world with a strong commitment in PE. A survey similar to ours was recently performed in Ontario [16]. The Ontario Committee found that many cancer centers have developed some of the recommended elements necessary to develop PE programs, but none of their centers encompassed all the core competences required (ibidem). In particular, the majority of Ontario centers treating cancer lack a strategic plan on PE, even though they all possess well-structured activities and tools for PE, a situation that substantially matches our findings in Italy.

The involvement of ICPEG, where thousands of cancer patients are treated every year, constitutes the remarkable strength of this study. The mission and vision of the centers participating in this survey are homogeneous, due to the predefined criteria required for the appointment by Ministry of Health [22]. Although this survey included a consistent sector of oncological care in Italy, this review refers to ICPEG, not to the entirety of oncology in Italy. As compared to hospitals or universities, the mission of CRCI is to cure and develop research activities only related to cancer, a situation that promotes more commitment. However, it is rather reassuring that responses were obtained from nearly all recognized CRCI throughout Italy.

This study has some limitations. While we took care in designing an appropriate survey, we may have missed some points that could integrate current information. In particular, we have not investigated specific issues like educational activities on clinical trials, on the process of delivering informed consent or on the role of research in improving PE programs [51]. Furthermore, the educational activity named “classes” is not a measurable activity devoted to training patients and/or caregivers in shared decision making [52], but, rather, hour long meetings with patients and caregivers on cancer topics held by HCWs where the conversation between patients and/or caregivers group and clinicians is encouraged/enhanced. Finally, due the complexity of this issue and the requirement of longitudinal assessments, we did not introduce measures of the effectiveness of PE activities in our survey.

Our survey suggests that, despite the widespread presence of PE activities in ICPEG, there are several areas of improvement that can further benefit PE programs and activities. One very important area of improvement regards the development of an institutionally based culture of PE organizations and programs that move from isolated, uncontrolled projects to a strategic approach. A second point of improvement is related to the role of patients in the development of PE; in fact, the role of the patient has been generally passive, while modern educational policies strive, for example, to maximize the contact between patients and HCWs [53]. Many other issues arise from our survey and need to be investigated such as the “expert patient” [54], his active role in the PE activities and in the design of the healthcare services [55].

Reconfiguring the healthcare workforce and developing more specific training for PE in HCWs curricula may be necessary. The definition of the most effective tools and activities remain an essential area of investigation, particularly considering the perils of illusory patient empowerment due to the uncontrollable patients’ searching for information on the Internet [56]. Finally, a recent analysis on the active role of patients in education/training reveals deficiencies both in the involvement of patients and in the quality of tools that critically evaluate this topic [57]. The evaluation of the impact of ongoing educational activities on the quality of cancer care using already existing instruments [42] represents a necessary step to be developed.

Implementation of a PE program in ICPEG and in all CRCI is an enormous challenge that requires effort and resources that should be carefully addressed, taking into account the unmet needs. In order to further enlarge the analysis, collect more data and propose new solutions at the national level, our project also aims to establish a group of dedicated professionals that promotes excellence models and standards to reach a high quality of PE education in cancer care. As an example, this group may prepare guidelines to help cancer centers, hospitals and teaching institutions more easily develop and measure educational services for their cancer patients.

## Conclusions

This work offers new insights on the situation of PE in the Italian CRCI. As this is the first step of the project, we hope that the results constitute a interesting framework to identify areas of development of next PE activities. A second step of the project will be to gather the points of view of the patients, HCWs and caregivers on the different activities. The development of these activities, together with more research on the effectiveness of the different activities will increase the culture of PE in Italy.

## Additional file

Additional file 1:
**Survey of patient education / empowerment (PE) Activities in Italian Cancer Care and Research Institutes (CRCI) Carried out in 2012. **(DOC 106 kb)

## Abbreviations

PE: Patient education
CRCI: Cancer Research and Care Institutes
ICPEG: Italian Cancer Patient Education Group
HCW: Healthcare worker
