# Supplementary material for: Making patient centered care a reality: a survey of patient educational programs in Italian Cancer Research and Care Institutes
Source: BMC Health Serv Res. 2015 Jul 30;15:298. doi: 10.1186/s12913-015-0962-5 (PMC4518580; doi:10.1186/s12913-015-0962-5)
Supplement: Additional file 1: — Survey of patient education / empowerment (PE) Activities in Italian Cancer Care and Research Institutes (CRCI) Carried out in 2012. (DOC 106 kb) [file 12913_2015_962_MOESM1_ESM.doc]

CENTRO DI RIFERIMENTO ONCOLOGICO

CRO

AVIANO

Istituto Nazionale Tumori - Aviano

Istituto di Ricovero e Cura a Carattere Scientifico di Diritto Pubblico (D.I. 31/07/1990)

Via Franco Gallini n° 2 - 33081 Aviano (PN) Italy - C.F.- P.I. 00623340932 - Tel. 0039-0434-659111 - Fax: 0039 0434 652182

Survey Questionnaire: the Scientific Directors of 13 Italian Cancer Research and Care Institutes (CRCI) were asked to complete the questionnaire, which was designed to investigate what type of Patient Education activities are carried out and how they are implemented.

Form N° ____

**SURVEY OF PATIENT EDUCATION / EMPOWERMENT (PE) ACTIVITIES
IN ITALIAN CANCER CARE AND RESEARCH INSTITUTES (CRCI)
CARRIED OUT IN 2012**

1. **Name of Institute __________________________**
2. **Name of Compiler __________________________**
3. **Role within the CRCI**

1⬜ Scientific Board Director

2⬜ Other, please specify ______________________________

1. **How many hospital beds in your institution?**

1 ⬜ Ward: no.___________________________

2 ⬜ Day Hospital: no._____________________

1. **How many new patients are hospitalized each year (approximately)?***Please refer to 2012*

1 ⬜ no._________________________

1. **How many people work in your institution?** *(full-time, fixed term, yearly contract, etc…)*

1 ⬜ no._________________________

1. **Does your institution have an institutional program for PE activities?**

1 ⬜ yes 2 ⬜ no

1. **Does your institution also have PE activities that are not centrally coordinated (e.g. those of individual departments)?**

1 ⬜ yes 2 ⬜ no

1. **With reference to question no. 8, do you believe this to be....**

1 ⬜ positive, why? __________________________________________________________________________________________________________________________________________________

2 ⬜ negative,why?

__________________________________________________________________________________________________________________________________________________

If you answered NO to both questions 7 and 8, you have completed the questionnaire; if you answered yes to at least one question, please go on in the next section.

**PATIENT EDUCATION ACTIVITIES**

For questions marked with an asterisk (*), you can specify more than one answer.

Please indicate what PE activities are offered at your CRCI and their characteristics

1. **Does you Institute organize informal conferences for patients on medical topics that are held by the healthcare staff,?**

1 ⬜ yes 2 ⬜ no

If you answered no, please go the question no.. 11

10a. Was a preliminary survey conducted to determine the topics of interest to patients?

1 ⬜ yes 2 ⬜ no

10b. If you answered yes, how was this survey conducted?

1⬜ self-compiled questionnaire

2⬜ interview

3⬜ focus group

4⬜ other (please specify)________________________________________

10c. (*) Which professionals are involved in the organization phase of this activity?

1 ⬜ doctors

2 ⬜ nurses

3 ⬜ administrative personnel

4 ⬜ psychologists

5 ⬜ librarians

6 ⬜ outside agency

7 ⬜ research personnel

8 ⬜ patients / volunteers

9 ⬜ other, _______________________________________

10d. (*)informal meetings/conferences with patients are held by:

1 ⬜ doctors

2 ⬜ nurses

3 ⬜ librarians

4 ⬜ outside agency

5 ⬜ research personnel

6 ⬜ other, _______________________________________

10e. How often are they held?

1 ⬜ once a week

2 ⬜ 2-3 times a month

3 ⬜ once a month

4 ⬜ 2-6 times a year

5 ⬜ other,__________________________________________

10f. (*) Where are they held?

1 ⬜ ward

2 ⬜ library

3 ⬜ meeting rooms

4 ⬜ clinic area

5 ⬜ other, __________________________________________

10g. (*)Which communication channels are used to inform patients of these informal conferences?

1 ⬜ posters hung in the Institute

2 ⬜ patient newsletter

3 ⬜ institutional web site

4 ⬜ healthcare workers (doctors, nurses, psychologists)

5 ⬜ volunteers

6 ⬜ various communications outside the institution (press releases, brochures, etc ..)

7 ⬜ institutional blog

8 ⬜other, __________________________________________

10h. Are patients involved in the organization phase of PE activities?

1 ⬜ yes 2 ⬜ no

10i. Are patients actively involved in the informal PE conferences (do they interact with the speakers)?

1 ⬜ yes 2 ⬜ no

10l. (*) How are patient evaluations on the informal conferences collected?

1 ⬜ self compiled questionnaires after the conferences

2 ⬜ interviews

3 ⬜ focus group

4 ⬜ not collected

5 ⬜ other, _____________________________________________

10m. Are the conferences based on the actual needs of the patients (as a result of questionnaires, interviews, etc…)?

1 ⬜ yes 2 ⬜ no

1. **Does your institute publish informative and educational materials for patients?**

1 ⬜ yes 2 ⬜ no

If you answered no, please go to question no. 12

11a. Was a survey conducted on the topics of interest to patients?

1 ⬜ yes 2 ⬜ no

11b. If so, how?

1⬜ self-compiled questionnaire

2⬜ interviews

3⬜ focus group

4⬜ other, _____________________________________________

11c. (*) Which professionals are involved in the production of these publications?

1 ⬜ doctors

2 ⬜ nurses

3 ⬜ administrative personnel

4 ⬜ psychologists

5 ⬜ librarians

6 ⬜ outside agency

7 ⬜ research personnel

8 ⬜ patients / volunteers

9 ⬜ other, _______________________________________

11d. How many are published every year?

1 ⬜ 1

2 ⬜ 2 - 5

3 ⬜ 6 - 10

4 ⬜ more than 10

11e. Who supervises the pubblication?

1 ⬜ external agency

2 ⬜ internal office

11f. If you answered internal office: which one?

1 ⬜ Public Relations Office

2 ⬜ Communications Office

3 ⬜ Library

4 ⬜ other, ______________________________________

11g. (*) Which communication channels are used to inform patients of the availability of informative materials?

1 ⬜ posters hung in the Institute

2 ⬜ patient newsletter

3 ⬜ institutional web site

4 ⬜ healthcare workers (doctors, nurses, psychologists)

5 ⬜ volunteers

6 ⬜ various communications outside the institution (press releases, brochures, etc ..)

7 ⬜ displays containing the informative material

8 ⬜ institutional blog

9 ⬜ other, ______________________________________

11h. Are patients involved in the production of informative materials?

1 ⬜ yes 2 ⬜ no

11i. (*) How is the published informative material made available?

1 ⬜ in literature holders throughout the institute

2 ⬜ information desks

3 ⬜ distributed by health care personnel

4 ⬜ Public Relations Office

5 ⬜ on the institute's website

6 ⬜ other, _____________________________________________

11l. (*) how are patient evaluations on the informative material collected?

1 ⬜ self-compiled questionnaire

2 ⬜ surveys

3 ⬜ focus group

4 ⬜ not collected

5 ⬜ other, _____________________________________________

11m. Are the publications based on the actual needs of patients (as a result of questionnaires, interviews, etc…)?

1 ⬜ yes 2 ⬜ no

1. **Is a Cancer information service for patients available in your institute?**

1 ⬜ yes 2 ⬜ no

If you answered no, go to question no. 13

12a. Was a survey conducted on the interest / usefulness of this service for patients?

1 ⬜ yes 2 ⬜ no

12b. If so, how?

1 ⬜ self-compiled questionnaire

2 ⬜ interviews

3 ⬜ focus group

4 ⬜ other, _____________________________________________

12c. (*) Which professionals are involved in this service?

1 ⬜ doctors

2 ⬜ nurses

3 ⬜ psychologists

4 ⬜ librarians

5 ⬜ outside agency

6 ⬜ research personnel

7 ⬜ patients / volunteers

8 ⬜ other, _______________________________________

12d. How many information desks are there in your institute?

1 ⬜ 1

2 ⬜ 2 - 3

3 ⬜ > 3

12e. (*) where are these cancer information desks?

1 ⬜ Entrance hall (or nearby)

2 ⬜ near the wards

3 ⬜ near the clinics

4 ⬜ day hospital area

5 ⬜ other, ____________________________________________

12e. How many hours a weeks is the information desk open to the public? (if you have more than one, please indicate the main desk)

1 ⬜ ≤ 10 hours

2 ⬜ 11 - 20 hours

3 ⬜ 21 - 30 hours

4 ⬜ > 30 hours

5 ⬜ undeterminable

12f. (*) Which communication channels are used to inform patients of this service?

1 ⬜ posters hung in the Institute

2 ⬜ patient newsletter

3 ⬜ institutional web site

4 ⬜ healthcare workers (doctors, nurses, psychologists)

5 ⬜ volunteers

6 ⬜ various communications outside the institution (press releases, brochures, etc ..)

7 ⬜ institutional blog

8 ⬜ other, please specify ______________________________________________________

12g. (*) How are patient evaluations on the cancer information service collected?

1 ⬜ questionnaires

2 ⬜ surveys

3 ⬜ focus group

4 ⬜ not collected

5 ⬜ other, _____________________________________________

12h. Is the activity based on the actual needs of the patients (as a result of questionnaires, interviews, etc…)?

1 ⬜ yes 2 ⬜ no

12i. Do the information desks keep track of their activities?

1 ⬜ yes 2 ⬜ no

12l. If so, how?

1 ⬜ file excel, access

2 ⬜ shared database, please specify, ______________________________

3 ⬜ institutional database

4 ⬜ other, _____________________________________________

12m. (*) if so, who is specially prepared to keep track of the activities at the information desks?

1 ⬜ Public Relations Office

2 ⬜ psychologists

3 ⬜ librarians

4 ⬜ external agency

5 ⬜ volunteers

6 ⬜ other, ____________________________

1. **Are training courses (CME and non CME) on topics such as communication, relationship with the patient, narration, offered to healthcare professionals?**

1 ⬜ yes 2 ⬜ no

If you answered no, please go to question no. 14

13a. (*) Which professionals are involved in the organization phase of this activity?

1 ⬜ doctors

2 ⬜ nurses

3 ⬜ psychologists

4 ⬜ education office

5 ⬜ librarians

6 ⬜ outside agency

7 ⬜ patients

8 ⬜ other, _______________________________________

13b. (*)which professional receive this training?

1 ⬜ doctors

2 ⬜ nurses

3 ⬜ administrative personnel

4 ⬜ psychologists

5 ⬜ librarians

6 ⬜ laboratory technician

7 ⬜ research personnel

8 ⬜ other, _______________________________________

13c. (*) What topics are addressed in the training programs?

1 ⬜ doctor-patient relationship (communication)

2 ⬜ narrative medicine

3 ⬜ counselling

4 ⬜ Neuro-linguistic programming (PNL) and other communication techniques

5 ⬜ humanization in healthcare

6 ⬜ ethics

7 ⬜ other, __________________________________

13c. How many courses on these topics are carried out every year?

1 ⬜ number:____________________

13d. Are patients involved in this training activity (mixed participation in courses by both healthcare staff and patients)?

1 ⬜ yes 2 ⬜ no

13e. (*) how are participant evaluations on courses carried out?

1 ⬜ self-compiled questionnaire

2 ⬜ surveys

3 ⬜ focus group

4 ⬜ not collected

5 ⬜ other, _____________________________________________

13h. Training courses are based on the actual needs of:

1 ⬜ patients

2 ⬜ staff

3 ⬜ other, ______________________________________________

1. **Are practical activities offered to patients (eg. Gymnastics, activities in the kitchen, meditation, make-up classes, etc ...)?**

1 ⬜ yes, please specify__________________________________________

2 ⬜ no

If you answered no, please go to question no. 15

14a. Was a survey conducted to determine the activities of interest to patients?

1 ⬜ yes 2 ⬜ no

14b. If so, how?

1 ⬜ self-compiled questionnaire

2 ⬜ interviews

3 ⬜ focus group

4 ⬜ other, _____________________________________________

14c. (*) which professionals are involved in the organization phase of this activity?

1 ⬜ doctors

2 ⬜ nurses

3 ⬜ administrative personnel

4 ⬜ psychologists

5 ⬜ librarians

6 ⬜ outside agency

7 ⬜ research personnel

8 ⬜ patients / volunteers

9 ⬜ other, _______________________________________

14d. (*) The activities with patients are lead by:

1 ⬜ doctors

2 ⬜ nurses

3 ⬜ psychologists

4 ⬜ librarians

5 ⬜ outside agency

6 ⬜ research personnel

7 ⬜ other, _______________________________________

14e. How often are they held?

1 ⬜ once a week

2 ⬜ 2-3 times a month

3 ⬜ once a month

4 ⬜ 2-6 times a year

5 ⬜ other,__________________________________________

14f. (*) Where are they held?

1 ⬜ ward

2 ⬜ library

3 ⬜ meeting rooms

4 ⬜ clinic area

5 ⬜ other, __________________________________________

14g. (*) Which communication channels are used to inform patients of these activities?

1 ⬜ posters hung in the Institute

2 ⬜ patient newsletter

3 ⬜ institutional web site

4 ⬜ healthcare workers (doctors, nurses, psychologists)

5 ⬜ volunteers

6 ⬜ various communications outside the institution (press releases, brochures, etc ..)

7 ⬜ institutional blog

8 ⬜ other, please specify ______________________________________________________

14h. Are patients involved in the organization phase of this activity?

1 ⬜ yes 2 ⬜ no

14i. How are patient evaluation on this activity collected?

1 ⬜ questionnaires

2 ⬜ surveys

3 ⬜ focus group

4 ⬜ not collected

5 ⬜ other, _____________________________________________

14l. Are these activities based on the actual needs of patients (as a result of questionnaires, surveys)?

1 ⬜ yes 2 ⬜ no

1. **Are activities dedicated to caregiver and family education carried out? (family members, relatives, caregivers)**

1 ⬜ yes 2 ⬜ no

IF you answered no, please go to question no. 16

15a. (*) if you answered yes, which activities are carried out?

1 ⬜ informative meetings on cancer and its treatment

2 ⬜ meeting on home care/what treatment can be done at home

3 ⬜ psychological support meetings

4 ⬜ other, _____________________________________________

15b. Was a survey conducted to determine the activities of interest to caregivers?

1 ⬜ yes 2 ⬜ no

15c. If so, how?

1 ⬜ self-compiled questionnaire

2 ⬜ interviews

3 ⬜ focus group

4 ⬜ other, _____________________________________________

15d. (*) which professionals are involved in the organization phase of this activity?

1 ⬜ doctors

2 ⬜ nurses

3 ⬜ administrative personnel

4 ⬜ psychologists

5 ⬜ librarians

6 ⬜ patients / volunteers

7 ⬜ outside agency

8 ⬜ research personnel

9 ⬜ other, _______________________________________

15e. (*) Activities for caregivers are lead by:

1 ⬜ doctors

2 ⬜ nurses

3 ⬜ psychologists

4 ⬜ librarians

5 ⬜ outside agency

6 ⬜ research personnel

7 ⬜ other, _______________________________________

15f. How often are they held?

1 ⬜ once a week

2 ⬜ 2-3 times a month

3 ⬜ once a month

4 ⬜ 2-3 times a year

5 ⬜ other,__________________________________________

15g. (*) Where are they held?

1 ⬜ ward

2 ⬜ library

3 ⬜ meeting rooms

4 ⬜ clinic area

5 ⬜ other, __________________________________________

15h. (*) Which communication channels are used to inform caregivers of these activities?

1 ⬜ posters hung in the Institute

2 ⬜ patient newsletter

3 ⬜ institutional web site

4 ⬜ healthcare workers (doctors, nurses, psychologists)

5 ⬜ volunteers

6 ⬜ various communications outside the institution (press releases, brochures, etc ..)

7 ⬜ institutional blog

8 ⬜ other, please specify ______________________________________________________

15i. Are caregivers involved in the organization phase of these activities?

1 ⬜ yes 2 ⬜ no

15l. How are caregiver evaluations on these activities collected?

1 ⬜ self-compiled questionnaire

2 ⬜ surveys

3 ⬜ focus group

4 ⬜ not collected

5 ⬜ other, _____________________________________________

15m. Are activities based on the acutal needs of the caregivers (as a result of questionnaires, surveys)?

1 ⬜ yes 2 ⬜ no

15n. Are activities based on the acutal needs of patients (as a result of questionnaires, surveys)?

1 ⬜ yes 2 ⬜ no

1. **Are self-help groups or other support groups present in your institutute?**

1 ⬜ yes, please specify what type of group ____________________________________

2 ⬜ no

If you answered no, please go to question no. 17

16a. Was a survey conducted to determine the needs of the patients?

1 ⬜ yes 2 ⬜ no

16b. If so, how?

1 ⬜ self-compiled questionnaire

2 ⬜ interviews

3 ⬜ focus group

4 ⬜ other, _____________________________________________

16c. (*) which professionals were involved in the organization phases of this activity?

1 ⬜ doctors

2 ⬜ nurses

3 ⬜ administrative personnel

4 ⬜ psychologists

5 ⬜ librarians

6 ⬜ patients / volunteers

7 ⬜ outside agency

8 ⬜ research personnel

9 ⬜ other, _______________________________________

16d. (*) The activities with patients are held by:

1 ⬜ doctors

2 ⬜ nurses

3 ⬜ librarians

4 ⬜ psychologists

5 ⬜ outside agency

6 ⬜ volunteers

7 ⬜ other, _______________________________________

16e. How often are they held?

1 ⬜ once a week

2 ⬜ 2-3 times a month

3 ⬜ once a month

4 ⬜ 2-3 times a year

5 ⬜ other,__________________________________________

16f. (*) Where are they held?

1 ⬜ ward

2 ⬜ library

3 ⬜ meeting rooms

4 ⬜ clinic area

5 ⬜ other, __________________________________________

16g. (*) Which communication channels are used to inform patients of group meetings?

1 ⬜ posters hung in the Institute

2 ⬜ patient newsletter

3 ⬜ institutional web site

4 ⬜ healthcare workers (doctors, nurses, psychologists)

5 ⬜ volunteers

6 ⬜ various communications outside the institution (press releases, brochures, etc ..)

7 ⬜ institutional blog

8 ⬜ other, please specify ______________________________________________________

16h. Are patients involved in the organization phase of this activity?

1 ⬜ yes 2 ⬜ no

16i. How are patient evaluations on these meetings collected?

1 ⬜ self-compiled questionnaire

2 ⬜ surveys

3 ⬜ focus group

4 ⬜ not collected

5 ⬜ other, _____________________________________________

16l. Are group activities based on the actual needs of the patients (as a result of questionnaires, surveys, etc…)?

1 ⬜ yes 2 ⬜ no

1. **Are volunteer organizations present in your institute?**

1 ⬜ yes 2 ⬜ no

17a. If so, is the relationship with the institute regulated by a formal agreement?

1 ⬜ yes 2 ⬜ no

17b. Does coordination exist between the volunteer organizations?

1 ⬜ yes 2 ⬜ no

17c. If so, what type?

1 ⬜ coordination by the institute

2 ⬜ autonomous coordination among the organizations

3 ⬜ other, ________________________________

17d. Do volunteers receive specialized training?

1 ⬜ yes 2 ⬜ no

17e. If so, the training program is planned/managed by:

1 ⬜ the volunteer organizations

2 ⬜ the institute

3 ⬜ jointly by the volunteer organizations and the institute

4 ⬜ other, ____________________________________________

1. **Are other PE activities conducted in your institute?**

1 ⬜ yes, please specify_______________________________________

2 ⬜ no

1. **(*) In your opinion, which topics should be addressed by a PE program in a Cancer Care and Re (CRCI)?**

1 ⬜ cancer screening

2 ⬜ cancer prevention (in general)

3 ⬜ advantages and disadvantages of cancer treatments

4 ⬜ appropriate use of cancer drugs outside the Institute (eg therapies at home)

5 ⬜ costs of cancer treatments

6 ⬜ decision aid tools to guide patients in making treatment choices

7 ⬜ communicate the news to patients, particularly negative news

8 ⬜ survivorship

9 ⬜ organizational aspects of care pathways (signs, timing and mode of outpatient clinics and hospital admissions, etc ...)

10 ⬜ clinical trials

11 ⬜ narrative medicine

12 ⬜ complementary medicine (CAM)

13 ⬜ communication and support to caregivers

14 ⬜ male and female fertility

15 ⬜ available community resources after hospital discharge

16 ⬜ other, _______________________________________

**Thank you for your collaboration!**

Date compiled _______________
